# Investigating the role of structural wall stress on aortic growth prognosis in acute uncomplicated type B aortic dissection

**DOI:** 10.21203/rs.3.rs-6569327/v1

**Published:** 2025-05-16

**Authors:** Minliang Liu, Yuhang Du, Hannah L. Cebull, Yuxuan Wu, Adam Mazlout, Asanish Kalyanasundaram, Rishika Agarwal, Hai Dong, Marina Piccinelli, John N. Oshinski, John A. Elefteriades, Rudolph L. Gleason, Bradley G. Leshnower

**Affiliations:** Texas Tech University; Texas Tech University; Emory University; Georgia Institute of Technology and Emory University; Georgia Institute of Technology and Emory University; Aortic Institute at Yale-New Haven Hospital, Yale University School of Medicine; Georgia Institute of Technology and Emory University; Emory University School of Medicine; Emory University; Georgia Institute of Technology and Emory University; Aortic Institute at Yale-New Haven Hospital, Yale University School of Medicine; Georgia Institute of Technology and Emory University; Emory University School of Medicine

**Keywords:** type B aortic dissection, aortic growth, optimal medical therapy, static determinacy, wall stress

## Abstract

**Objective:**

False lumen expansion is a major factor that determines long-term survival of uncomplicated type B aortic dissection (TBAD). The objective of this study was to investigate whether structural wall stress distributions computed from patient-specific acute TBAD geometries can be used to predict aortic growth rates.

**Methods:**

Three-dimensional (3D) computed tomography angiography (CTA) of 9 patients with acute uncomplicated TBAD were obtained at initial hospital admission and at their most recent follow-up visits. Patient-specific structural wall stress distributions were computed from the initial baseline CTA using a forward penalty method. Spatially varying blood pressure distributions, derived from computational fluid dynamics (CFD) simulations informed by patient-specific transthoracic echocardiography (TTE) and blood pressure (BP) measurements, were incorporated into the forward penalty stress analysis. Aortic growth rates were quantified and visualized within the 3D TBAD geometries using the initial baseline and follow-up scans. Linear mixed-effects regression analyses were performed to evaluate the spatial correlations between biomechanical markers (structural wall stress, wall shear stress, and pressure) and aortic growth rates.

**Results:**

Utilizing initial baseline CTA, TTE, and BP data, the forward penalty analyses revealed hemodynamic and structural mechanics insights of acute uncomplicated TBADs. The linear mixed-effects model indicated that the fixed-effect association between structural wall stress and aortic growth rate distributions was statistically significant (p=0.039), which demonstrated that aortic segments experiencing high wall stress exhibited rapid growth. Fixed-effect associations were not significant when predicting growth rate using wall shear stress (p=0.86) or pressure (p=0.61) distributions. Significant Pearson correlation coefficients (p<0.05) were observed between structural wall stress and aortic growth rate in all patients.

**Conclusion:**

High structural wall stress was associated with regions of high aortic growth rates, while false lumen thrombosis was associated with low wall stress. Structural wall stress derived from the forward penalty approach may be a novel predictor of aortic growth rate and failure of optimal medical therapy in acute TBAD.

## 1. INTRODUCTION

Type B Aortic Dissection (TBAD) is a lethal disease with an incidence of 4 per 100,000 patients per year [1]. TBAD occurs when a tear develops in the inner lining (intimal layer) of the aorta, causing the layers of the aortic wall to separate (dissect), creating “true” and “false” lumens. Acute TBAD patients are classified as “complicated” based upon the presence of organ malperfusion or aortic rupture; otherwise, they are considered “uncomplicated” [2]. Acute complicated TBAD carries a high mortality rate and is optimally treated with emergent thoracic endovascular aortic repair (TEVAR) [3]. Acute uncomplicated TBAD has been traditionally treated with optimal medical therapy (OMT) consisting of aggressive anti-hypertensive and anti-impulse medications and surveillance imaging. OMT provides excellent short-term survival (83–100%), but is less effective as the dissection ages, with long-term survival rates of 48–66% and overall intervention-free survival rates of < 50% [4–7]. Recent retrospective reports have demonstrated improved survival with TEVAR compared to OMT in acute uncomplicated TBAD, although no Level I evidence currently exists [8]. The ability to accurately predict aortic growth would allow the identification of patients at high risk for OMT failure that may benefit from early TEVAR and improve long-term TBAD survival.

Previous work has identified demographic and anatomic risk factors associated with OMT failure, aortic growth, and reduced survival in TBAD. These factors include diabetes mellitus, end-stage renal disease, maximum descending aortic diameter ≥ 45 mm, primary intimal tear > 10 mm, the proximity of the primary intimal tear to the left subclavian artery, a patent or partially thrombosed false lumen, and a false lumen diameter > 22 mm [3, 9–18]. Aortic diameter has been the only reproducible risk factor that has emerged from these studies, but diameter alone has been shown to be inadequate in predicting the risk of aortic growth, dissection or rupture in aortic disease [19–22]. Over the past decade, our group and others have investigated alternative factors that may play an integral role in determining aortic growth in TBAD, namely false lumen hemodynamics and the biomechanical properties of the false lumen wall [23, 24]. Computational fluid dynamics (CFD) and four-dimensional (4D) flow magnetic resonance imaging (MRI) have been used to assess hemodynamics in TBADs and evaluate patients’ risks [25–32]. However, these simulation and imaging techniques primarily provide data related to blood flow (e.g., blood pressure, velocity, wall shear stress, etc.) within the aorta. They do not offer insights into the structural biomechanics of the aortic wall, where the structural stress is several orders of magnitude higher than wall shear stress. To investigate the role of structural wall stress, conventional fluid-structure interaction (FSI) analysis [33, 34] can be employed which provides data on both hemodynamics and structural biomechanics. However, conventional two-way FSI methods are labor-intensive and time-consuming, with high computational costs. Completing a single patient’s FSI analysis can take weeks [19, 35, 36], which makes it impractical for clinical prognostic applications that require rapid feedback to clinicians. Moreover, while two-way FSI with patient-specific tissue material properties is theoretically feasible, determining these patient-specific properties also requires an expensive iterative computation process [37–39], which greatly reduces the practical value of this complex strategy. In addition, using material parameters from one patient for another can introduce significant uncertainties in the simulations.

Fortunately, our group and others have recently shown that the aortic wall structural stress can be correctly computed by using the principle of static determinacy without knowing patient-specific material properties. This has led to the development of a simple, computationally efficient method known as the forward penalty approach [40–42], achieved by enforcing an artificially stiff treatment to the aortic wall. In this study, we employed a similar approach to compute structural wall stress distributions in patients with acute uncomplicated TBAD. By utilizing static determinacy, the need for two-way solver communication is eliminated, which results in a structural stress computation workflow with significantly reduced computational time. Patient-specific, spatially-varying blood pressure distributions, derived from CFD simulations based on inlet flow conditions obtained via clinical transthoracic echocardiograms, were used to compute structural wall stress distributions. Additionally, 3D heatmaps of aortic growth rates were computed using CT scans obtained at initial diagnosis and follow-up. The purpose of this investigation was to determine whether the spatial distribution of structural wall stress correlated with the spatially-varying aortic growth rate in patients with acute uncomplicated TBAD. Developing a tool that could reliably predict aortic growth in acute uncomplicated TBAD could impact the treatment (e.g. OMT vs TEVAR) in these patients and improve survival.

## 2. METHODS

### 2.1. Image segmentation and meshing

**Figure 1:**
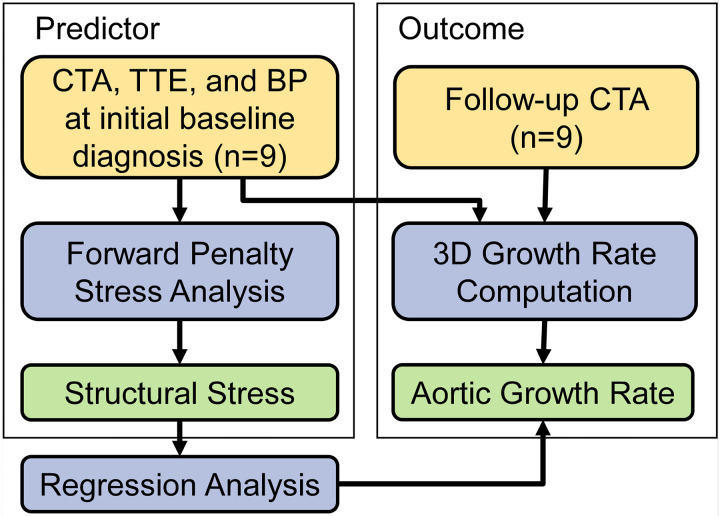
Workflow of study design. CTA: computed tomography angiography; TTE: transthoracic echocardiogram; BP: blood pressure measurement. Forward penalty analysis was performed to compute structural wall stress distribution. Aortic growth rate was quantified on three-dimensional (3D) TBAD geometries. The correlation between stress and growth rate was quantified by using regression analysis.

**Figure 2:**
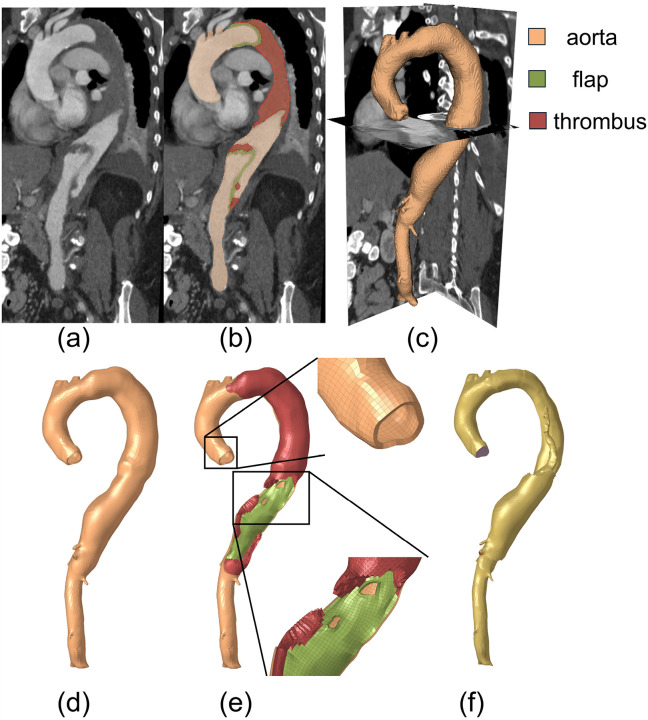
CT image segmentation and mesh generation of a representative patient-specific TBAD geometry. Segmentation of aorta (a), dissection flap (b), and thrombosis (c) from baseline CT scans. Generation of computational meshes of the aortic wall (d), dissection flap, and thrombus (e) and blood-occupied fluid domains (f).

Figure 1 shows the workflow of the overall study design. With approval from the institutional review board (IRB), we retrospectively collected clinical computed tomography angiography (CTA) images, transthoracic echocardiographic data, blood pressure, and demographics of 9 patients who presented with acute uncomplicated TBAD and were initially treated with OMT. These data were acquired at the initial admission following the diagnosis of aortic dissection. Additionally, to quantify the rate of aortic diameter expansion, the most recent follow-up CT scans were collected for each patient. The CTA, echocardiogram, and blood pressure were collected as part of standard routine clinical care. The CTA images had an averaged in-plane voxel resolution of 0.715mm, and a z-axis voxel resolution of 1mm. Since ECG gating was not available, we assumed the CTA images corresponded to the systolic phase. Patient-specific 3D geometries of the dissected thoracoabdominal aorta, including brachiocephalic artery, left common carotid artery, left subclavian artery, celiac artery, superior mesenteric artery, the dissection flap, and intraluminal thrombosis, were semi-automatically segmented from the baseline CTA scans of initial hospital admission by the ‘grow from seed’ tool available in the 3D Slicer software. The aorta, dissection flap, and thrombosis (if present) were segmented separately. Figure 2 (a)~(c) illustrates the 3D segmented geometries of a representative patient. The segmented dissection flap incorporates tears/fenestrations, which allowed accurate geometric representations for the subsequent simulations. Watertight surfaces were extracted from the aorta, flap and thrombosis segmentations by the ‘model maker’ tool in 3D Slicer.

**Figure 3:**
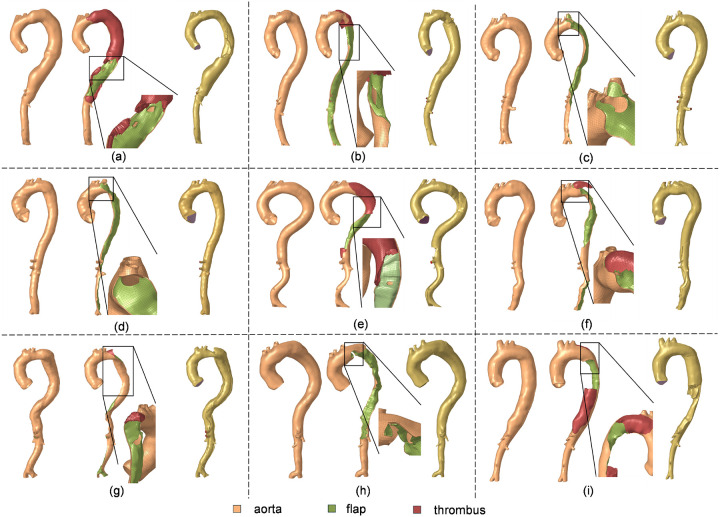
Computational meshes of the solid and fluid domains of all 9 patients involved in this study. Zoomed views of the primary intimal tear are shown. (a) ~ (i): P1 to P9. Thrombosis is present in P1, P2, P5, P6, P7, and P9.

Following the segmentation process, the surfaces representing aorta, flap and thrombosis segmentations were utilized to generate computational meshes in Altair Hypermesh for fluid and solid computations (see Fig. 2(d)~(f)). This involved the following steps: (1) The CT-derived surfaces of the aortic wall and dissection flap were re-meshed primarily using 2D quadrilateral elements, creating conforming 2D surface meshes at the interfaces of the aortic wall and flap. Following the mesh independent analysis from a previous study [43], an element size of 2mm was employed for the surfaces meshes. (2) Solid meshes for the aortic wall and dissection flap were generated by offsetting the 2D surface meshes in surface normal directions. Due to the limited resolution of the CT scans, wall thickness may not be accurately measured. An inward offset of 1mm was applied to the true lumen, followed by an outward offset of 1mm for the outer false lumen wall. Consequently, the true lumen was offset twice, resulting in a wall thickness of 2mm, while the false lumen and dissection flap were offset once, leading to a wall thickness of 1mm. These thickness values are based on our group’s experimental work with TBAD tissue specimens [24]. This approach ensured that the combined thickness of the dissection flap and false lumen wall equaled the intact aortic wall thickness, which naturally simulates the wall thickness in acute aortic dissections when the freshly dissected flap separates from the false lumen wall. To enhance the accuracy of solid mechanics computations, the true and false lumen walls and dissection flap were meshed using 4 layers of 3D hexahedral elements (C3D8H). (3) Thrombosis, if present, was then meshed using 3D tetrahedral elements and stitched to the aortic wall meshes with nodal connectivity. This led to approximately 146,707 elements in the solid domain mesh for each patient. (4) Subsequently, the fluid domain occupied by blood was meshed, which is comprised of 8 boundary layers (3D hexahedral elements) and a core (3D tetrahedral elements). To determine the CFD element size, we conducted a mesh independence analysis and analyzed the CFD-predicted peak velocity in a dissection flap fenestration of a representative geometry using various maximum element sizes (4, 3, 2, 1mm). The mesh at smaller branches and fenestrations are refined by using a factor of 0.25 relative to the maximum element size. The mesh independence test indicated that a CFD mesh consisting of approximately 388,911 elements, with a maximum element size of 2 mm is sufficient. The fluid and solid domain meshes conformed at their interface, allowing for data exchange between the solid and fluid domains. Figure 3 illustrates the solid and fluid domain meshes for the 9 patients included in this study. On average, the segmentation took about 6 hours and meshing took about 12 hours for a human expert.

### 2.2. Structural wall stress computation using the forward penalty approach

We employed a clinically-accessible forward penalty approach to compute structural wall stress distributions on the aortic wall using the baseline CT-derived TBAD geometries. The validity of this forward penalty approach is warranted by the fact that the aortic wall is approximately statically determinate [40], which allows structural stress to be readily computed from image-derived geometries without the need for identifying patient-specific material properties or modeling realistic tissue deformations.

**Figure 4:**
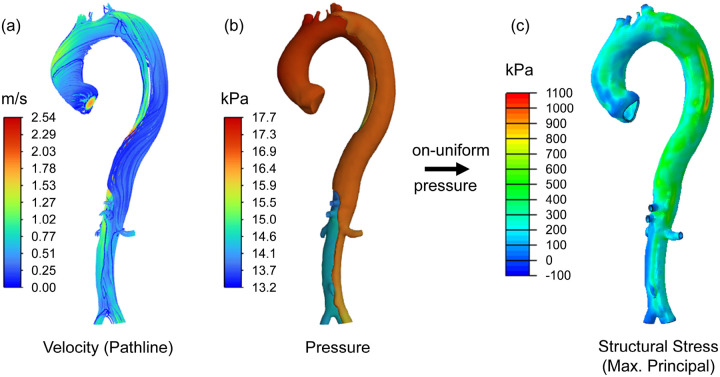
Forward penalty stress computation workflow for stress computation: (a) flow pathlines color-coded with velocity; (b) pressure contour; (c) structural wall stress distribution computed by enforcing an artificially stiff material as penalty treatment to the aortic wall.

As depicted in Fig. 4, forward penalty stress computations were conducted utilizing the CTA at initial diagnosis of each patient, involving the following steps: (1) Patient-specific computational fluid dynamics (CFD) simulations were performed in ANSYS Fluent: For patient P2, P3, P4, P6, and P7, the peak aortic valve velocity measured by echocardiography served as the inlet flow condition for the ascending aorta, with a turbulence 1/7 power law velocity profile [44]. Because echocardiography data was not available for P1, P5, P8 and P9, a constant flow rate of 25 L/min was applied at the aorta inlet, representing a population-averaged physiological flow rate during peak systole [45]. Windkessel models with parameters calculated using Murray’s law [46] were applied at each outlet to match the patient’s systolic blood pressure measurement obtained during the initial hospital admission. (2) The nonuniform blood pressure distribution in the true and false lumen wall was extracted from the CFD simulation and mapped to the solid domain mesh using an in-house 3D interpolation code. (3) Subsequently, structural wall stress distributions in the true and false lumen wall, as well as the dissection flap, were derived from structural finite element analysis (FEA) in Abaqus using the spatially-varying pressure distribution computed in the CFD simulation as the loading condition. Using the principle of static determinacy of the aortic wall [40], structural wall stress was computed by enforcing an artificially stiff material (Young’s modulus 5×10^5^ kPa) as penalty treatment to the aortic wall, ensuring that the displacement remains negligibly small (on the order of 10^−3^ millimeter), which eliminated the need for patient-specific material properties or realistic aortic wall deformations. If intraluminal thrombosis is present in the patient-specific TBAD anatomy, a Young’s modulus of 2.5×10^4^ kPa is assigned to the thrombus such that the ratio of stiffness between aortic wall and thrombus is kept constant, as established by Joldes et al.[47]

TBAD anatomy may be distinct from that of a healthy or aneurysmal aorta due to the presence of the dissection flap. To validate the forward penalty stress computation approach for TBAD geometries, we compared the stress field obtained using the forward penalty method with that computed via a fully coupled FSI approach. The mean absolute percentage error (MAPE) of the two stress fields is 7.57%. The details of this validation are provided in the Appendix.

### 2.3. Quantification of aortic growth rate

**Figure 5:**
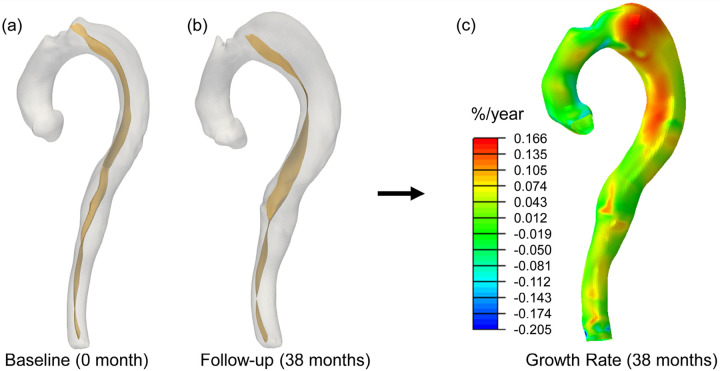
Initial baseline (a) and follow-up TBAD geometries (b) are used to compute the heatmap of aortic growth rate (c).

For each patient, the follow-up TBAD geometry was segmented from most-recent follow-up CTA and meshed with 2D elements, following the same procedure described in section 2.2. In this study, we computed the aortic growth rate at every node of the TBAD meshes using the initial and most recent follow-up CTA scans, which allowed visualization of the growth rate as a heatmap distribution. To generate this heatmap, the baseline TBAD geometry at initial diagnosis was meshed with quadrilateral elements by using a mesh parameterization approach [48]. Thus, the mesh consists only of quadrilateral elements and is topologically equivalent to the lateral surface of a cylinder. It consists of 50 nodes along each circumference and 200 circumferential layers along the axial direction, forming a structured mesh. The baseline and follow-up meshes were pre-aligned using the ICP algorithm [49] to remove rigid body motions. Subsequently, the baseline geometry was morphed to match the follow-up geometry through a large deformation diffeomorphic metric mapping framework [50] implemented in the Deformetrica software [51]. This nonlinear registration process established point-to-point mesh correspondence [52] between the baseline and follow-up TBAD geometries, which enabled the calculation of displacement field between the two geometries. Subsequently, logarithmic strain, which quantifies the percentage aortic growth in the circumferential direction, was calculated from the displacement field. The aortic growth rate was then determined by dividing the strain by the number of years between the baseline and follow-up CTA. This work presents growth rate heatmaps in %/year, as mm/year is not a meaningful metric at localized spatial points. To improve clinical interpretability, the spatially averaged aortic diameter growth rate (in mm/year) was calculated for each patient by averaging the diameter change rates across the descending aorta. The workflow for quantifying the aortic growth rate is illustrated in Fig. 5.

### 2.4. Statistical Analysis

To assess the 3D spatial correlation between the distribution of structural wall stress and the heatmap of aortic growth rate, we first aligned the structural wall stress and growth rate distribution data and established point-to-point correspondence, i.e., the subsequent spatial correlation analysis requires that a spatial point on the stress distribution is aligned with the same spatial point on the growth rate heatmap. To achieve this, the stress distribution was registered using the ICP algorithm [49] onto the structured mesh where growth rate data was stored; therefore, stress and growth data share the same 3D coordinates. To enhance data analysis and visualization, the following sampling method was employed. Using the structured mesh from the growth rate analysis, each patient’s aorta was divided into 50 equally sized regions along the axial direction, with each region comprising 4 circumferential layers. Based on this partitioning, the descending aorta, from the left subclavian artery to the aortic bifurcation, consists of 38 regions. Subsequently, the averaged maximum principal stress and aortic growth rate can be calculated for each region, which resulted in 38 stress-growth rate data points for each patient. This data sampling method allowed for the quantification and visualization of the stress-growth rate spatial correlation.

To account for inter-patient variability, linear mixed-effects regression analysis was then conducted to investigate the relationship between structural wall stress and aortic growth rate using a total of 342 data points (38 regions each patient, 9 patients). In a linear mixed-effects model, the slope and intercept are separated into fixed-effects (slope *β*_1_ and intercept *β*_0_) and random-effects (slopes ***b***_1*m*_ and intercept ***b***_0*m*_, where ***m*** = **1,2**, …, **9**is patient ID). The fixed-effects remain constant across all patients, which characterize the overall association between stress and growth rate in the patient population (n=9); the random-effects are adjusted to describe the stress-growth rate correlation for each individual patient and can vary from patient to patient. To test whether a statistically significant correlation exists between stress and growth rate, F-test was performed on the fixed-effect slope *β*_1_ in the linear mixed-effects model, with the null hypothesis being that the fixed-effect *β*_1_ slope is zero. A p-value smaller than 0.05 was considered statistically significant to reject the null hypothesis and indicate a non-zero fixed-effect slope *β*_1_. Estimate and 95% confidence interval (CI) of the standard deviation of the random-effect slopes ***b***_1*m*_ are also reported in the linear mixed-effects model, which quantify the inter-patient variability. Pearson correlation coefficient and its p-value were used to quantify the stress-growth correlation for individual patients. For comparison with structural stress performance, we also employed the systolic wall shear stress and systolic blood pressure as predictors and performed linear mixed-effects regression analysis.

## 3. RESULTS

### 3.1. Study sample

The demographic information of the patients involved in this study is summarized in Table 1. Among the 9 patients analyzed for outcomes, the median follow-up period was 3.18 years. Spatially-averaged growth rates of the descending aorta were calculated for each patient using the method described in Section 2.4, resulting in a median spatially-averaged descending aorta growth rate of 1.84 mm/year. Table 1 also presents the median and interquartile ranges of demographic variables such as age, gender, and the number of visceral vessels originating from the true and false lumens.

### 3.2. Hemodynamics

**Figure 6:**
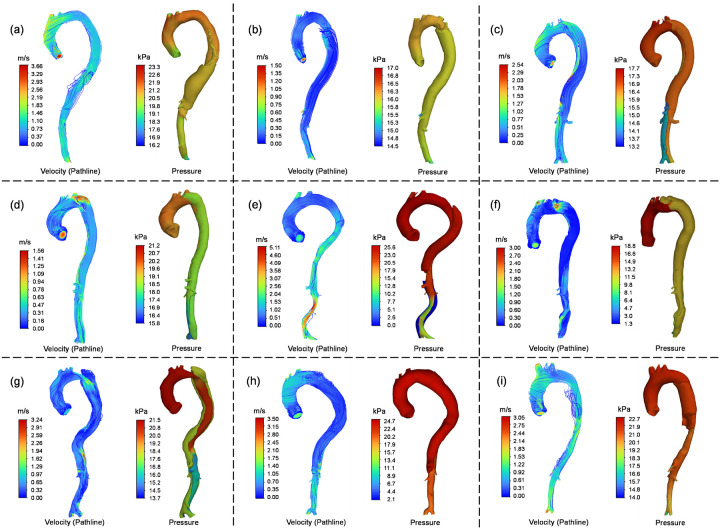
Pressure distribution and pathline results of the TBAD patients. The pathlines were color-coded with the velocity of blood flow. (a) ~ (i): P1 to P9

Hemodynamic outcomes, including pressure distribution and pathlines of TBAD blood flow, were computed from the forward penalty analyses, as depicted in Fig. 6. The pathline results were color-coded according to the velocity of blood flow. Blood pressure is one of the primary contributors to structural wall stress, according to the law of Laplace [8, 53]. Utilizing Windkessel outlets, the simulated systolic blood pressure in the ascending aorta was consistent with clinically measured systolic blood pressure from the patient’s right arm. Pressure differences between the true and false lumens stemmed from unique geometric characteristics of TBAD geometries, such as the size and location of the primary intimal tear and the presence of intraluminal thrombosis.

CFD simulations showed average pressures of 18.36 kPa in the true lumen and 17.60 kPa in the false lumen. The false lumen exhibited a more uniform pressure distribution, likely due to its typically larger cross-sectional area compared to the narrower true lumen, which led to an observable pressure drop. A paired-sample t-test comparing average pressures between the true and false lumens yielded a p-value of 0.4285, indicating no statistically significant difference. Combined with the pressure contour results, these findings suggest that pressure distributions between the true and false lumens are highly patient-specific.

### 3.3. Contours of structural stress and aortic growth rate

**Figure 7:**
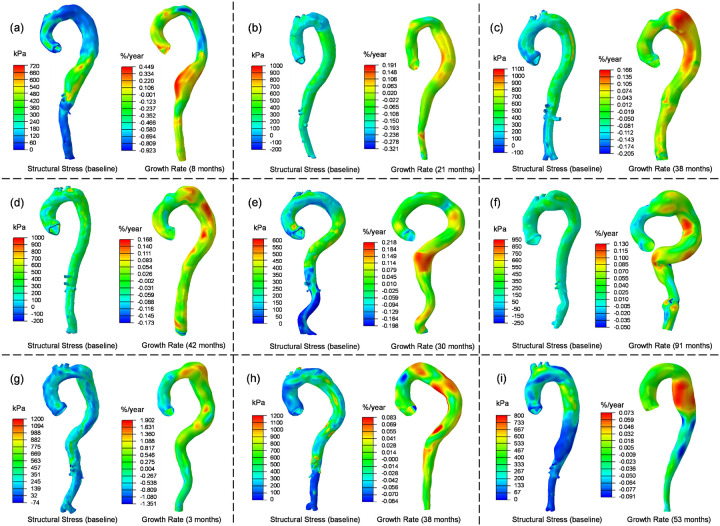
Structural wall stress distributions computed using geometry at initial baseline diagnosis and heatmaps of the corresponding patients’ aortic growth rates. (a) ~ (i): P1 to P9.

Structural wall stress fields computed based on the initial baseline CT scans were compared to the heatmap of the aortic growth rate, as depicted in Fig. 7. Results from the analysis of the 9 patients revealed a general correlation between structural stress and growth rate: aortic segments experiencing high structural wall stress demonstrated rapid aortic growth. This correlation was particularly evident in patients P1, P3, P5, P7, P8, and P9. For instance, the distal descending aorta of P1 exhibited the highest wall stress in the baseline geometry, and this same region experienced rapid aortic growth (~ 30%/year) over the follow-up period (8 months).

**Figure 8:**
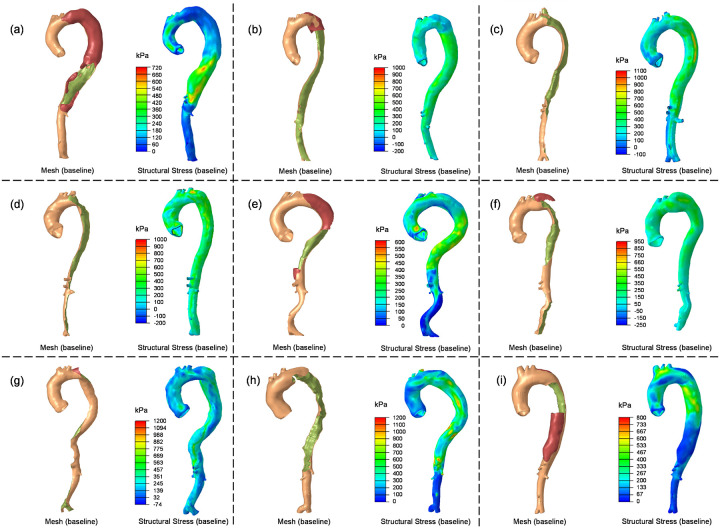
Comparison of aorta geometries at initial baseline diagnosis and structural wall stress distributions of the TBAD patients. (a) ~ (i): P1 to P9.

In four patients (P1, P2, P5 and P9), negative aortic growth rates were observed in segments of descending aorta due to a reduction in aortic diameter in the follow-up scan compared to the baseline scan. This was due to the formation of thrombus and subsequent reduction in false lumen diameter. Comparison between TBAD geometries and stress distributions (Fig. 8) revealed that regions with thrombosed false lumens exhibited much lower structural wall stress compared to other regions exposed to high blood pressure. This finding suggests that thrombus may serve a protective role against high structural wall stress.

In contrast to the high structural stress observed in the true and false lumens, the dissection flap experienced significantly lower stress levels, typically below 50 kPa. This is attributed to the fact that the dissection flap does not bear load and is subjected to pressures from both the true and false lumens.

### 3.4. Quantification of spatial correlation between structural stress and aortic growth rate

**Figure 9:**
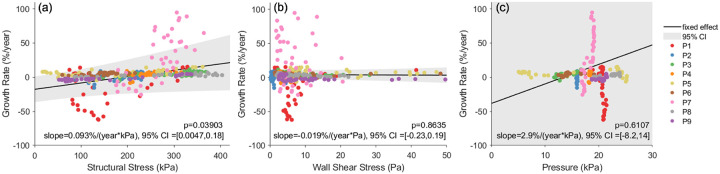
(a) Linear mixed-effects regression analysis demonstrates a significant fixed-effect association between structural wall stress at initial baseline diagnosis and aortic growth rate (p=0.039). The same analysis indicated insignificant fixed-effect associations using wall shear stress (b) and blood pressure (c) distributions. CI: confidence interval.

Linear mixed-effects regression analysis was performed which revealed a positive fixed-effectassociation between structural wall stress and growth rate considering all 9 patients. As shown in Fig. 9,F-test indicated that the fixed-effect slope *β*_1_was statistically significant (p = 0.039) to reject the nullhypothesis that *β*_1_ is zero. The estimated *β*_1_ is 0.093%/(year·kPa) with a 95% CI of [0.0047, 0.18]%/(year·kPa). For comparison, the systolic wall shear stress and systolic blood pressure were also tested as predictors in linear mixed-effects models. Wall shear stress led to a near-zero, negative fixed-effect *β*_1_ (−0.019%/(year·Pa)), while pressure resulted in a positive fixed-effect *β*_1_ (2.9%/(year·kPa)). However, the 95% CI for pressure was wide ([−8.2, 14] %/(year·kPa)) due to highly-patient specific spatial pressure distributions. The results indicated that the fixed-effect associations were not statistically significant for wall shear stress (p = 0.86) or blood pressure (p = 0.61).

## 4. DISCUSSION

### 4.1. Forward penalty stress analysis as a potential predictive tool

In this study, we investigated the relationship between the structural wall stress distribution and aortic growth rate in acute uncomplicated TBAD using a forward penalty simulation workflow and 3D aortic growth heatmaps. Linear mixed-effects regression suggested that TBAD growth is significantly associated with the structural stress exerted on the aortic wall. We observed a positive association between high wall stress regions and high growth rate regions, indicating that high structural wall stress could serve as a predictor of locations prone to rapid aortic growth. To the best of our knowledge, this is the first study to investigate the role of structural wall stress in the progression of acute uncomplicated TBAD. Biomechanical simulations [36], such as the forward penalty stress analysis employed in this study, can offer clinicians personalized insights into anatomical sites vulnerable to rapid aortic growth during TBAD progression. This patient-specific information adds to the current risk factor profile and allows for tailoring the treatment to the individual patient. For instance, a patient presenting with an acute uncomplicated TBAD with a proximal descending aortic diameter of 40 mm and a forward penalty stress analysis showing high structural wall stress in the proximal descending aorta could undergo TEVAR to reduce structural wall stress in this high-risk segment of the false lumen. This may prevent the development of a large aneurysm requiring a high-risk open procedure in the chronic phase and improve long-term survival. Conversely, structural stress data could inform the treatment of a patient with a proximal descending aortic diameter of 40 mm and low structural wall stress, who could be treated with OMT alone, and avoid the risks of stroke, spinal cord ischemia, vascular injury, and the adverse effects of aortic stiffening that occur with TEVAR. Due to the small sample size (9 patients) in this study, it may be challenging to establish a universal structural stress criterion for distinguishing high-risk from low-risk patients for aortic expansion and development of false lumen aneurysms. Whether such universal stress criterion exists warrants further investigations.

Conventional fully-coupled FSI simulations, which integrate fluid and solid mechanics solvers, provide high-fidelity biomechanical results but are often computationally prohibitive for complex geometries like patient-specific aortic dissection, requiring days to weeks to complete for a single patient. [35, 54]. As a result, fully-coupled FSI analysis is typically restricted to a very small number of patients in the literature [36, 55, 56] and may not be feasible for larger simulation cohorts due to its time-consuming nature.

Moreover, these simulations often encounter convergence issues due to the high nonlinearity in geometry and material models. Heavy domain knowledge and skills are required to tackle the convergence errors. In contrast, our use of a statically-determinant forward penalty model in this study enabled rapid computation of structural wall stress. The simulation run time is usually under 20 minutes on a desktop PC. Due to the use of static determinacy in the solid mechanics setup, this approach is robust against convergence problems. This novel, rapid forward penalty model affords the possibility of clinicians incorporating patient-specific data into their decision-making in determining the optimal therapy (OMT vs. TEVAR) for patients with acute uncomplicated TBAD.

### 4.2. Structural wall stress vs. wall shear stress

In this study, we computed structural wall stress, which should not be confused with wall shear stress (WSS). WSS refers to the frictional force exerted by flowing blood on the vessel wall and is often considered a predictor of plaque formation in arterial atherosclerosis. This flow-induced frictional force is mechanical sensed by endothelial cells and mechanotransduced into a biochemical signal that regulates vascular functionality [57]. Changes or disturbances in WSS patterns can lead to alterations in cellular proliferation, thrombosis, and inflammation within the vessel wall. However, in the context of TBAD progression, the role of WSS may require further investigation, as the endothelium may not exist in the acutely dissected false lumen wall where aortic expansion predominantly occurs. Our findings also did not reveal a significant association between WSS and aortic growth rate. Furthermore, WSS typically ranges 1 ~ 10 Pa [58], whereas the structural wall stress computed in this study is several orders of magnitude larger, typically around 300 kPa (Fig. 7). Structural wall stress arises from tension induced by blood pressurization, which may serve as a more relevant biomechanical regulator of aortic expansion in acute TBAD.

### 4.3. Modeling assumptions and limitations

In this work, the two-way interaction between aortic wall and blood flow was not modeled. Instead, we assumed steady-state condition in the fluid domain and computed structural stress by using a clinically more accessible forward penalty approach. This modeling scheme was chosen due to the following considerations: (1) While fully coupled FSI analysis can provide a more detailed hemodynamic assessment, including pulsatile blood pressure, it is computationally expensive, often requiring days to weeks for a single patient simulation, making it impractical for clinical applications. Most fully coupled FSI studies on TBAD are limited to a very small sample size [56]. Our goal in this study is to adopt a computationally efficient and clinically applicable approach to investigate the role of structural stress in TBAD progression. (2) A recent study [59] with a larger patient cohort has demonstrated that blood pressure fields computed from CFD simulations are predictive of TBAD clinical outcomes. Furthermore, it has been shown that CFD-predicted true and false lumen pressure fields exhibit minimal differences when compared to those obtained from two-way FSI simulations [60, 61]. (3) The mechanical properties of the dissection flap, as well as the true and false lumen walls, are highly nonlinear and patient-specific, yet largely unknown from routine CT imaging, which provides data from only a single cardiac phase. Accurately modeling nonlinear structural mechanics would require incorporating pressure-induced prestress and growth-driven residual stress. These unknown factors, along with their complex interplay, must be fully incorporated into the modeling before an accurate FSI-based hemodynamic and structural mechanics assessment could be achieved. However, comprehensively incorporating these effects from routine single-phase CT images is not feasible. (4) It has been previously demonstrated that transmurally averaged structural stress can be accurately computed within minutes using the static determinacy approach, without requiring patient-specific material properties [40–42]. Additionally, this transmurally averaged stress is independent of prestress and residual stress [40]. As shown in the Appendix, the stress field obtained using the forward penalty method and that computed via a fully coupled FSI approach are nearly identical (MAPE 7.57%).

The wall thickness of the TBAD geometry may be difficult to obtain from clinical CT images due to limited resolutions and partial volume effect. In this study, we modeled acute TBAD and utilized the following thicknesses in the 3D TBAD model: (1) 2 mm for the true lumen wall, (2) 1 mm for the false lumen wall and dissection flap. These thickness values are consistent with our group’s experimental work with TBAD tissue specimens [24].

Due to the retrospective nature of this study, we acquired baseline and follow-up CT images at various intervals and calculated the temporally-averaged growth rate of the outer surface of the aorta. It is important to recognize that false lumen growth is non-linear, can vary between patients, and can vary significantly between the acute and chronic phases [62]. For instance, P7 exhibited a growth rate of approximately 50% per year, whereas most other patients’ growth rates were around 5% per year. This discrepancy may be attributed to the short time interval between the baseline and follow-up CT scans for P7 (3 months). Although the time interval could influence the calculation of growth rates, our observation that high-stress regions were associated with rapid growth regions remains unaffected. In future work, the aortic growth rate may be analyzed separately for the true and false lumen.

This study serves as a pilot investigation into the role of structural stress in TBAD progression; therefore, a limited number of patients (n = 9) was analyzed. In future work, we aim to further expand our forward penalty stress analysis by incorporating a larger patient cohort. With a larger dataset, we may establish a universal structural stress threshold to identify patients at high risk of rapid aortic expansion. Moreover, additional biomechanical markers may be identified to predict the risk of aneurysmal formation and the need for early endovascular intervention in acute uncomplicated TBAD. By further incorporating accurate constitutive description [63, 64], growth and remodeling theories [65, 66], in vivo material properties [37–39, 67], and failure modeling [68, 69] into the modeling framework, it can be anticipated that the rate and location of aortic growth in acute uncomplicated TBAD may be predicted by forward penalty simulations using information available in the initial baseline CT images.

## 5. CONCLUSION

The results of this work indicated that the forward penalty workflow can be used to compute patient-specific structural wall stress distributions of acute uncomplicated TBADs in a computationally efficient manner. Our regression analysis of structural stress distributions and growth rate heatmaps revealed a significantly positive association between high wall stress regions and areas with high growth rates, which suggested that elevated structural wall stress may serve as a novel predictor of anatomic locations at risk for rapid aortic growth. Future investigations will expand upon these findings by including a larger cohort of patients to establish a structural stress-based criterion for predicting rapid aortic growth.

## Supplementary Files

This is a list of supplementary files associated with this preprint. Click to download.

Appendix.docx

## Figures and Tables

**Figure 10:**
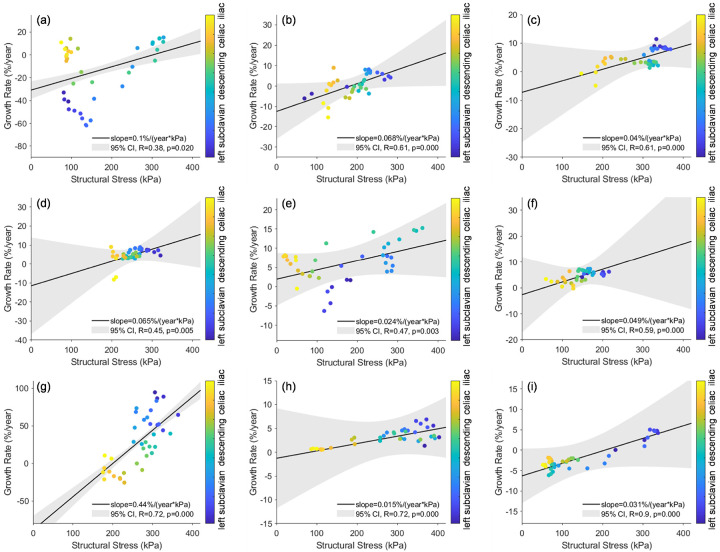
Structural wall stress distribution at initial baseline diagnosis and aortic growth rate of individual patients. (a) ~ (i): P1 to P9. The stress- growth rate data points are color-coded by spatial location from proximal to distal aorta. The regression lines were obtained by using both fixed- and random-effects in the linear mixed-effects model. CI: confidence interval; R, p: Pearson correlation coefficient and p-value.

**Table 1.** Patient demographics in the study population. The number of arteries includes celiac, superior mesenteric, inferior mesenteric, left renal, and right renal arteries. For numerical variables, the median, 25th and 75th percentiles are reported. Percentage is reported for dichotomic variables.

Age at the time of diagnosis (years)	62 [49.5, 67.5]
Gender (% male)	44.44
Follow-up period (years)	3.18 [1.19, 4.83]
Hypertension (%)	88.89
Beta-blocker (%)	22.22
Diabetes mellitus (%)	0
Stroke or transient ischaemic attack (%)	0
Dyslipidemia (%)	0
End-stage renal disease (%)	0
Smoking (%)	44.44
Congestive heart failure (%)	0
Marfan syndrome (%)	0
Number of arteries arising from true lumen	3[2, 4.5]
Number of arteries arising from false lumen	2 [0.5, 4]
Spatially-averaged aortic growth rate (mm/year)	1.84 [0.32, 2.32]

## Data Availability

The data that support the findings of this study are available from the corresponding author upon reasonable request.
